# Maintenance and Distribution of Epithelial Stem/Progenitor Cells after Corneal Reconstruction Using Oral Mucosal Epithelial Cell Sheets

**DOI:** 10.1371/journal.pone.0110987

**Published:** 2014-10-24

**Authors:** Takeshi Soma, Ryuhei Hayashi, Hiroaki Sugiyama, Motokazu Tsujikawa, Shintaro Kanayama, Yoshinori Oie, Kohji Nishida

**Affiliations:** Department of Ophthalmology, Osaka University Graduate School of Medicine, Suita, Japan; Osaka University Graduate School of Medicine, Japan

## Abstract

We assessed the maintenance and distribution of epithelial stem/progenitor cells after corneal reconstruction using tissue-engineered oral mucosal cell sheets in a rat model. Oral mucosal biopsy specimens were excised from green fluorescent protein (GFP) rats and enzymatically treated with Dispase II. These cells were cultured on inserts with mitomycin C-treated NIH/3T3 cells, and the resulting cell sheets were harvested. These tissue-engineered cell sheets from GFP rats were transplanted onto the eyes of a nude rat limbal stem cell deficiency model. Eight weeks after surgery, ocular surfaces were completely covered by the epithelium with GFP-positive cells. Transplanted corneas expressed p63 in the basal layers and K14 in all epithelial layers. Epithelial cells harvested from the central and peripheral areas of reconstructed corneas were isolated for a colony-forming assay, which showed that the colony-forming efficiency of the peripheral epithelial cells was significantly higher than that of the central epithelial cells 8 weeks after corneal reconstruction. Thus, in this rat model, the peripheral cornea could maintain more stem/progenitor cells than the central cornea after corneal reconstruction using oral mucosal epithelial cell sheets.

## Introduction

Corneal epithelial stem cells are located in the basal layer of the limbus, which is the narrow transition zone between the cornea and the conjunctiva [1], [2]. The limbal epithelium is a reservoir for replacing corneal epithelial cells that are normally continuously lost from the corneal surface [3]. Severe corneal diseases, such as Stevens–Johnson syndrome, or chemical burns destroy the limbus and cause limbal stem cell deficiency (LSCD). In these cases, corneal epithelial cell sources are exhausted, the peripheral conjunctival epithelium invades inwardly, and the corneal surface becomes enveloped by vascularized conjunctival scar tissue, which results in corneal opacification that leads to severe visual impairment [4], [5].

In cases of severe LSCD, we and others recently demonstrated the successful application of constructs involving ex vivo expansion of autologous oral mucosal epithelium [6]–[8]. This method averts the risks of immune rejection and long-term immunosuppression, and thus offers clinical advantages over conventional allogeneic corneal transplantation [9]. We have performed transplantation of oral mucosal epithelial cell sheets in over 20 patients. For these patients, corneal transparency was restored and postoperative visual acuity remained improved for 2–8 years, whereas abnormal corneas were successfully reconstructed using conventional allogeneic transplantation in only 20–30% of patients for 2–3 years [10], [11].

Cultured oral mucosal epithelial cell sheets contain stem/progenitor cells, as demonstrated by colony-forming assays (CFAs) and immunohistochemistry [6], [7]. Clinically successful long-term reconstruction after cell sheet transplantation suggests that these transplanted stem/progenitor cells are maintained in vivo postoperatively [12]–[14]. Although a few studies have investigated the existence of stem/progenitor cells in reconstructed cornea [15],[16], this has yet to be established. Thus, in this study, we assessed the maintenance and distribution of epithelial stem/progenitor cells after corneal reconstruction using oral mucosal epithelial cell sheets in a rat model.

## Materials and Methods

### 2.1. Primary culture of GFP rat oral mucosal epithelium

**Figure 1:**
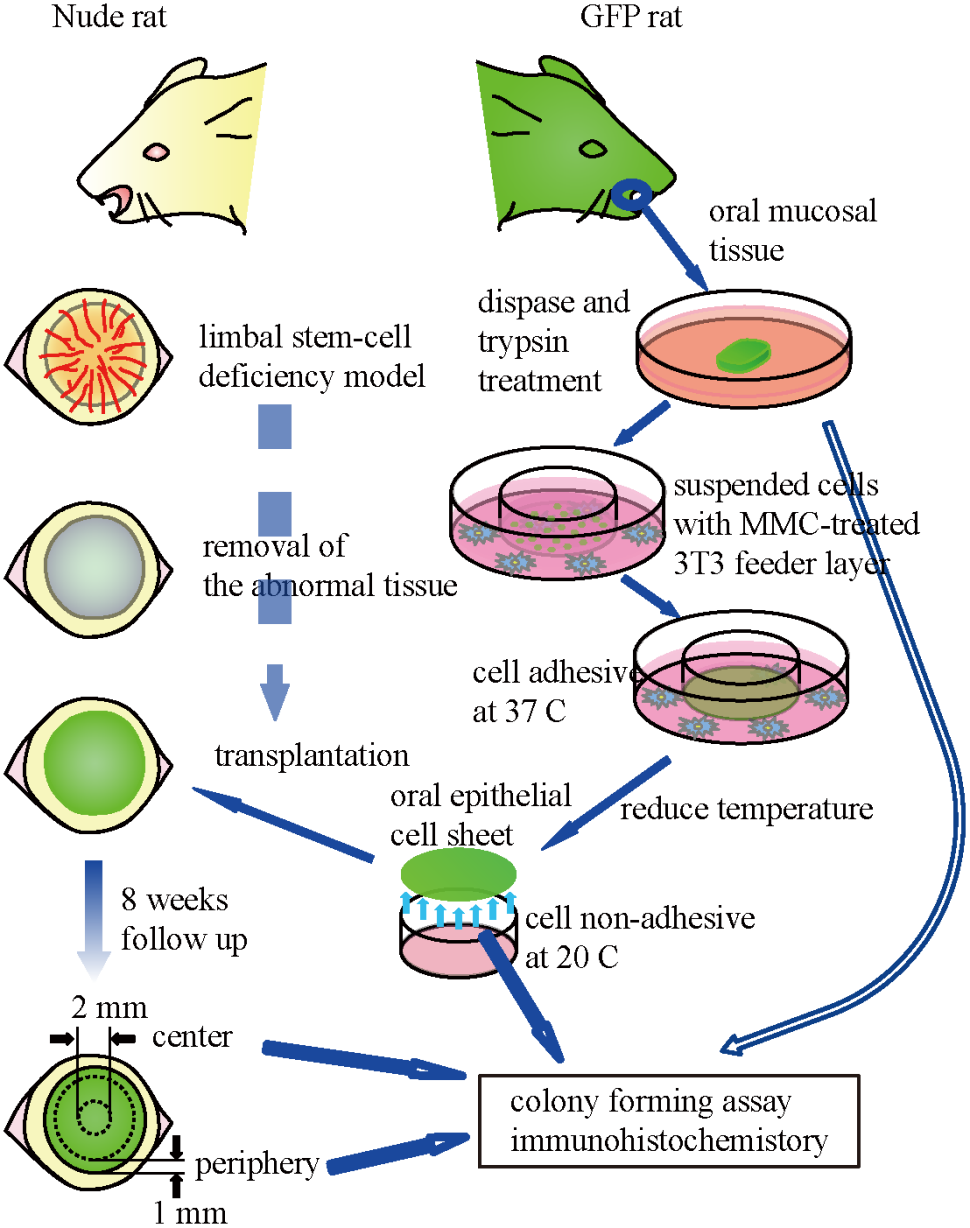
Transplantation strategy of cultured cell sheets fabricated from GFP rat oral mucosal epithelial cells onto the eyes of a nude rat limbal stem cell deficiency model.

Animals were treated in accordance with the ARVO Statement for the Use of Animals in Ophthalmic and Vision Research. Our experimental procedures were approved by the Committee for Animal Research of Osaka University Graduate School of Medicine. We created cultured cell sheets fabricated from GFP rat oral mucosal epithelial cells and transplanted it onto the eyes of a nude rat limbal stem cell deficiency model (Fig. 1). Oral mucosal biopsy specimens (2 mm radius) were excised from 4 green fluorescent protein (GFP) rats (“green rat CZ-004,” SD TgN (act-EGFP) OsbCZ-004; Japan SCL, Inc., Shizuoka, Japan). Each rat weighed 200 g. Anesthesia was induced by intraperitoneal administration of ketamine hydrochloride (25 mg/kg) and xylazine hydrochloride (10 mg/kg). Biopsy specimens were incubated at 4°C for 4 h with Dispase II (Roche Diagnostics GmbH, Mannheim, Germany) and treated with trypsin/EDTA solution (Invitrogen, Carlsbad, NM) at room temperature for 20 min. Cell suspensions were cultured on temperature-responsive culture inserts (CellSeed Inc., Tokyo, Japan) at an initial density of 4×10^5^ cells/23-mm insert along with mitomycin C (MMC)-treated NIH/3T3 cells that were separated by these cell culture inserts in the keratinocyte culture medium (KCM) (Dulbecco’s modified Eagle’s medium [DMEM]/F12 [3∶1] supplemented with 10% fetal bovine serum [Japan Bio Serum, Hiroshima, Japan], 0.5% Insulin–Transferrin–Selenium [ITS; Invitrogen, Carlsbad, CA], 10 µM isoproterenol [Kowa, Tokyo, Japan], 2.0×10^−9^ M triiodothyronine [MP Biomedicals, Aurora, OH], 0.4 µg/mL hydrocortisone succinate [Wako, Osaka, Japan], and 10 ng/mL EGF [R&D Systems, Minneapolis, MN]) [6]. Five days later, oral epithelial cells achieved confluence. After an additional 5–7 days of culture, the resulting cell sheets were harvested by reducing the culture temperature to 20°C for 30 min.

### 2.2. Transplantation of cultured oral epithelial cell sheets

A limbal stem cell deficiency model was generated in one eye each of anesthetized nude rats (F344/NJcl-rnu/rnu) by excising all corneal epithelial and limbal tissues (N = 4). Three weeks after surgery, conjunctival scar tissue with some neovascularization covered the entire corneal surface and invaded into the stroma. Prior to cell sheet transplantation, the conjunctivalized ocular surface was surgically removed to re-expose the corneal stroma. Tissue-engineered culture cell sheets, fabricated ex vivo from GFP rat oral mucosal epithelial cells, were harvested and transplanted over the stromal bed. For healing protection, tarsorrhaphy was performed after transplantation. Antibiotics and steroids were topically applied postoperatively 3 times daily. The eyes were carefully observed using a slit lamp biomicroscope and a fluorescence stereomicroscope. Eight weeks after surgery, the rats were sacrificed with an overdose of anesthetic agent (pentobarbital), and their eyes were enucleated for histology examinations and CFA.

### 2.3. Immunofluorescence and histological examinations

Oral mucosal epithelium, tissue-engineered epithelial cell sheets, and reconstructed corneas were assessed using immunofluorescence examinations. Cryosections (thick, 10 µm) were treated with 5% bovine serum albumin (BSA) in 50 mM Tris-buffered saline (TBS; pH 7.2) containing 0.4% Triton X-100 at room temperature for 60 min. The sections were then incubated overnight at 4°C with primary antibodies diluted with 1% BSA in PBS containing 0.4% Triton X-100. Primary antibodies included mouse monoclonal anti-p63 (4A4; Santa Cruz Biotechnology Inc., Santa Cruz, CA), mouse monoclonal anti-K14 (CKB1; Abcam, Tokyo, Japan), rabbit polyclonal anti-CD31 (Abcam), and Alexa Fluor 495 or 555-labeled secondary antibodies (Jackson ImmunoResearch Laboratories Inc., West Grove, PA) were used. All sections were counterstained with Hoechst 33342 and observed under a fluorescence microscope (Axiovert 200; Carl Zeiss, Oberkochen, Germany). Transplanted corneal sections were also conventionally stained with hematoxylin and eosin (HE) and observed under a light microscope (BX50; Olympus, Tokyo, Japan).

### 2.4. CFA

We used CFA to assess if there were putative progenitor cell populations in the biopsied, cultured, and transplanted cells. Primary cells isolated from GFP rat oral epithelial tissues were seeded in untreated 6-well culture plates for CFAs. Secondary cells isolated from tissue-engineered cell sheets by trypsin digestion were also used for CFAs. Transplanted epithelial cells from reconstructed corneas were also used for CFAs as follows. Corneoscleral tissues were excised from enucleated eyes 8 weeks after transplantation. As assessed using the fluorescence stereomicroscope, all of the epithelium that covered a cornea expressed GFP. The central areas (diameter, 2 mm) and peripheral areas (width, 1 mm) of the corneas were excised and treated with Dispase II at 37°C for 1 h. Epithelial cells were then separated and treated with 0.25% trypsin/EDTA solution at 37°C for 20 min to create single cell suspensions. Cells at a density of 3×10^3^ cells/well were used for CFAs for biopsied, cultured, and transplanted epithelial cells along with MMC-treated 3T3 feeder cells in 6-well culture plates. After 12 days in culture, the cells were fixed and stained with rhodamine B. The colony-forming efficiency (CFE) of primary, cultured, and transplanted cells was determined by dividing the number of colonies per well by the total number of seeded cells in each well (N = 4, duplicates used for each sample).

### 2.5. Statistical Analysis

Results are presented as mean ± SEs. Data were analyzed using t tests; p<0.05 was considered statistically significant. All statistical analyses were carried out using JMP version 9.0.3.

## Results

**Figure 2:**
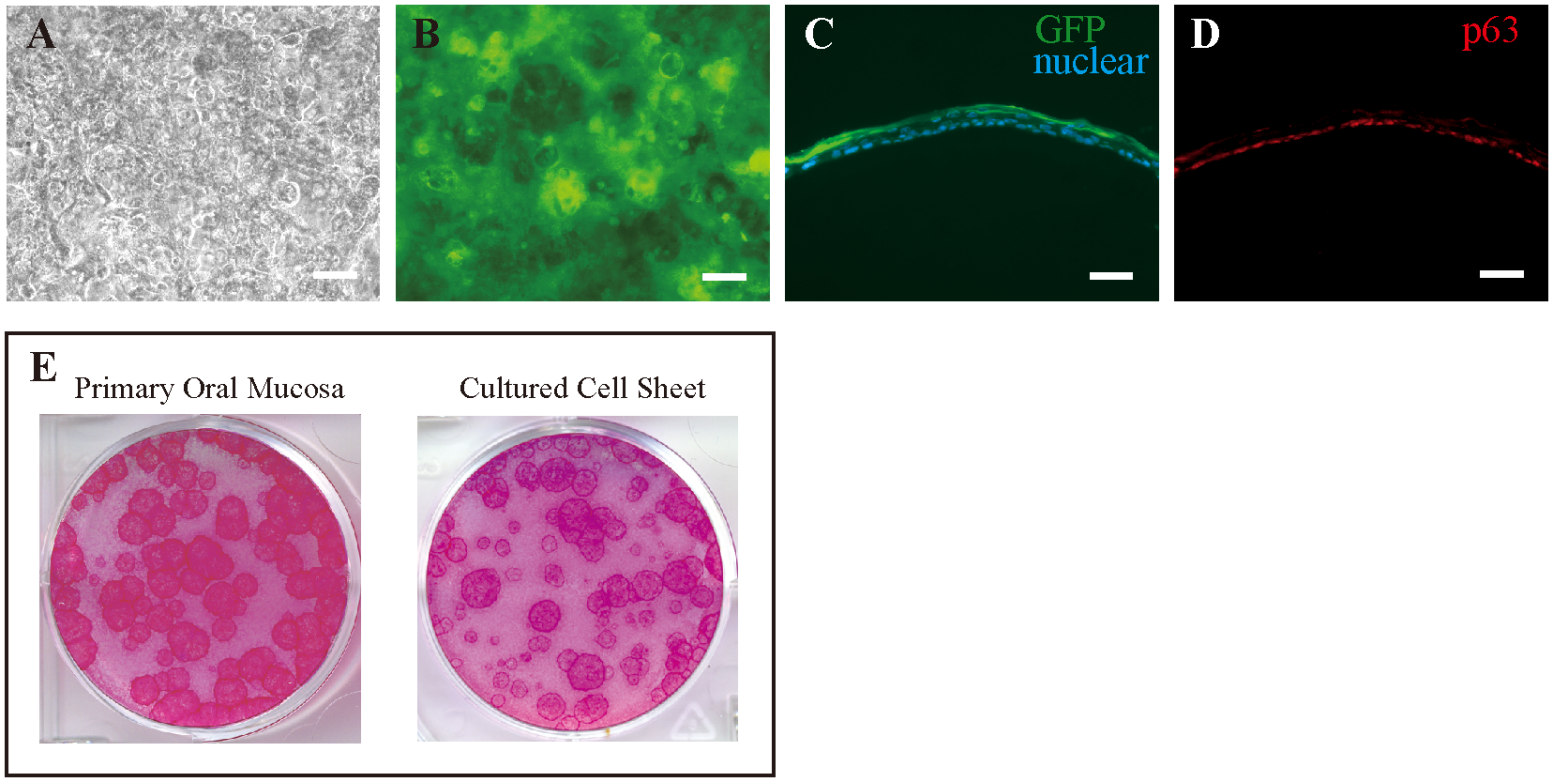
Successful generation of transplantable epithelial cell sheets from GFP rats. (A) Phase-contrast image of oral mucosal epithelial cells. (B) Fluorescence microscopy showing GFP-positive cells from a donor GFP rat. (C) Cross-section of a GFP-positive cell sheet. (D) Basal cells of cultured epithelial cell sheets express p63. (E) Both primary oral mucosa and cultured cell sheets contained sufficient numbers of progenitor cells. Scale bars = 200 µm (A, B) and 50 µm (C, D).

To monitor the cell fates of transplanted cell sheets, we prepared epithelial cell sheets fabricated from GFP rats. Phase contrast microscopy showed that oral mucosal epithelial cells obtained from the GFP rats proliferated and became stratified after culture for 10 days (Fig. 2A). These cells showed tight, dense packing on culture inserts as well as a cobble stone-like cell morphology, and fluorescence microscopy showed that all of these cells were GFP positive (Fig. 2B). Epithelial cell sheets were also evaluated on sections. The epithelial cell sheets were well stratified with 2–3 layers of GFP-positive epithelial cells (Fig. 2C). Immunohistochemistry results showed that the basal cells of cultured epithelial cell sheets expressed p63, a putative epithelial stem cell marker (Fig, 2D). The mean (±SE) CFE values for cells from the primary oral mucosa and cultured cell sheets were 3.17±0.67% and 2.12±0.68%, respectively, and both primary oral mucosa and cultured cell sheets contained sufficient numbers of progenitor cells (Fig. 2E).

**Figure 3:**
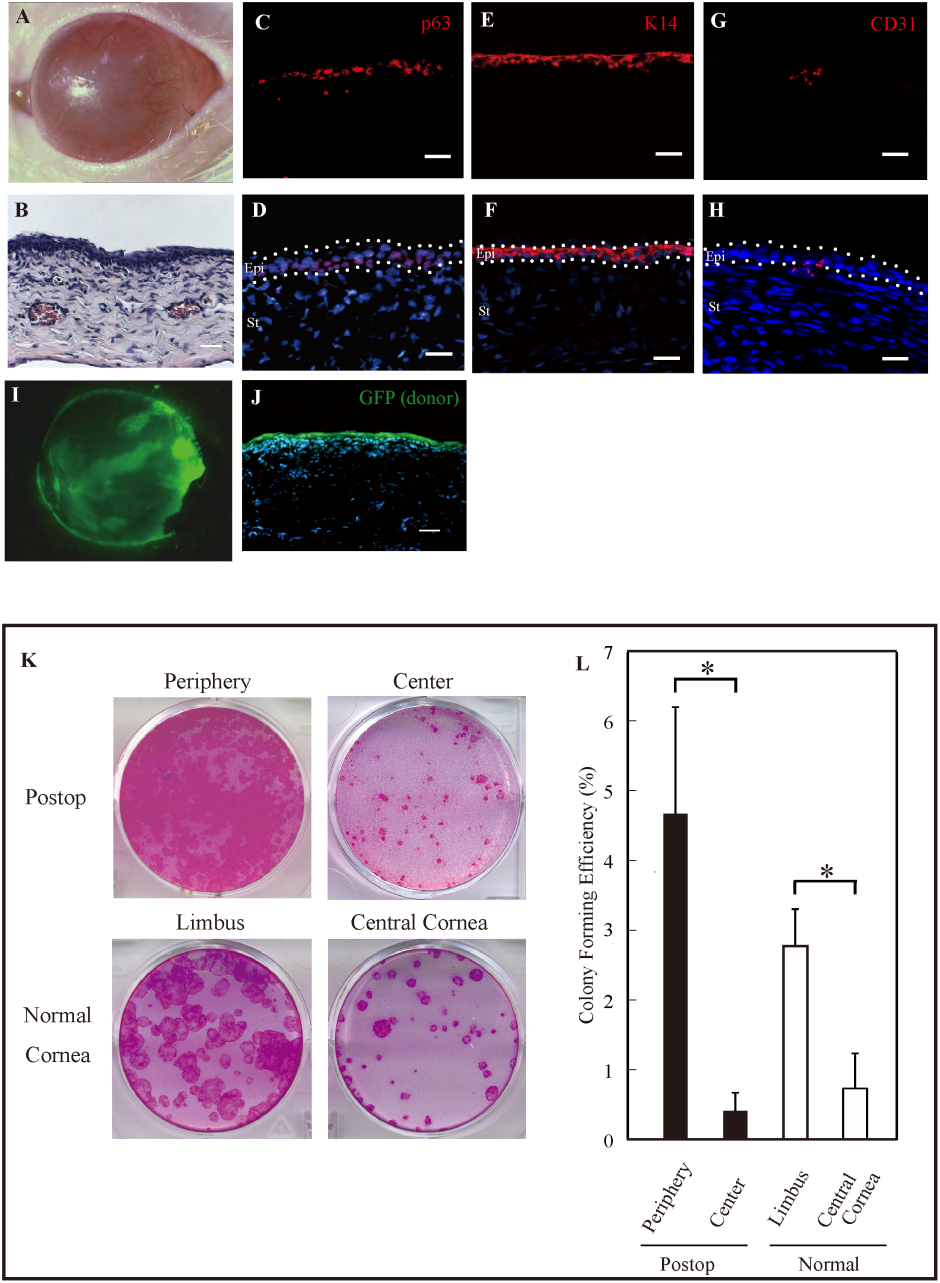
Eight weeks after transplanting cultured cell sheets prepared from GFP rats. (A) Slit lamp photograph of a reconstructed nude rat cornea. (B) HE staining showing that the reconstructed cornea was covered with 3–4 epithelial cell layers. (C, D) Immunostaining results showing that a reconstructed cornea still expressed p63 in the basal layers. (E, F) K14 was expressed in all epithelial layers after cell sheet transplantation. (G, H) The expression of CD 31 was observed in the basal epithelial layer and the superficial stroma in the peripheral cornea. (I) Corneal surface completely covered with GFP-positive epithelial cells. (J) Fluorescence microscopy showing that surviving GFP-positive epithelial cells were on the surface of a transplanted cornea. (K) CFAs for peripheral and central epithelial cells prepared from a postoperative eye and limbal and central epithelial cells prepared from a normal cornea. (L) The mean CFE values for the peripheral cells removed from the transplanted cornea was significantly greater than that for the central cell, which was also observed in a normal cornea (N = 4, *p<0.05). Epi, epithelium; St, stroma. Scale bars = 20 µm (C, D, E, F, G, H) and 25 µm (B, I).

Eight weeks after transplantation, a slit lamp photograph showed that the surface of a cornea was completely covered with epithelium (Fig. 3A). HE staining of a transplanted corneal section showed that 3–4 cell layers of epithelium were reconstructed (Fig. 3B). Immunostaining results showed that a transplanted cornea expressed p63 in the basal layers and K14 in all epithelial layers (Fig. 3C, 3D, 3E, 3F). In the peripheral region obvious neovascularization was observed in slit lamp examination (Fig. 3A). The presence of neovascularization was confirmed by the immunohistochemistry of CD31 (Fig. 3G, 3H). The transplanted cell sheet successfully reconstructed the corneal surface, and the entire cornea was covered by the GFP-positive multilayered epithelium (Fig, 3I). Cross-section analysis also demonstrated that the GFP-positive cells were on a reconstructed cornea (Fig. 3J).

To compare the distributions of putative epithelial stem/progenitor cells in the reconstructed corneas with those in the normal corneas, we used CFAs for epithelial cells harvested from both peripheral and central areas of the corneas in cell sheet-transplanted eyes (Fig. 3K). The reconstructed cornea contained colony-forming cells, and the mean (±SE) CFE values for the peripheral and central epithelial cells removed from the transplanted corneas were 4.67±1.53% and 0.41±0.26%, respectively, which results were significantly different (p = 0.036; Fig. 3L). In normal rats, the mean CFE value for the peripheral cornea was significantly higher than that for the central cornea (2.78±0.53% vs. 0.73±0.50%, p = 0.024; Fig. 3L).

## Discussion

The aim of this study was to examine the maintenance and distribution of epithelial stem/progenitor cells after corneal reconstruction using oral mucosal epithelial cell sheets in a rat model. Our findings indicate that cultivated oral mucosal epithelial cell sheet survives and contains putative epithelial stem/progenitor cells after transplantation. In addition, epithelial stem/progenitor cells are maintained abundantly in peripheral cornea, which shows a similar distribution pattern of stem cells to normal eyes.

When we performed autologous cell sheet transplantation in LSCD patients, it was difficult to distinguish whether the transplanted cell sheets survived, because these sheets were derived from the patients themselves. To resolve this problem, we used cultured cell sheets fabricated from GFP rats in this study. Using this method, the presence of GFP-positive cells after cell sheet transplantation in reconstructed corneas established that the transplanted cell sheets survived postoperatively.

We successfully generated the oral mucosal epithelial cell sheets from GPF rat. The epithelial cell sheets expressed p63 in the all of the basal layers and contained sufficient numbers of progenitor cells. Based on these results, we determined that these rat oral mucosal epithelial cell sheets were of sufficient quality for our subsequent transplant experiments [17].

After cell sheet transplantation, the entire cornea was covered by the GFP-positive cells, and they also expressed p63 in the basal layer and K14 in all epithelial layers. These findings suggested that the transplanted cell sheets survived and retained stem/progenitor cells for at least 8 weeks postoperatively. These results strongly support a hypothesis that stem/progenitors cells can survive after this treatment, which results in the long-term success of transplantation of cultured oral mucosal cell sheets in LSCD patients.

The CFE values for the peripheral epithelial cells removed from the reconstructed cornea were significantly higher than that for the central cells. This non-uniform pattern over the cornea indicates that, after transplantation, stem cell precursors are predominantly in the peripheral cornea compared with the central cornea. This suggests that stem cell progenitor maintenance is not cell autonomous, possibly owing to different microenvironments. It is interesting that this is also true in normal corneas [18].

The mechanism underlying the enrichment of progenitors in peripheral regions is not entirely clear. It may be due to the peripheral vascularization. The origin of the cell sheet is oral mucosa, a vascularized tissue, inducing neovascularization. We and others reported cultivated oral mucosal cell sheet contains highly level of angiogenic factors, such as basic Fibroblast growth factor or Thrombospondin 1, compared to cultivated corneal epithelial cell sheet [19], [20]. In fact, peripheral neovascularization occurs frequently in human clinical application of oral mucosal cell sheet transplantation reported by us and others [7], [12]–[14]. These neovascularization might contribute to maintain the stemness. For example, in the bone marrow, several reports have shown that vascular endothelial cells regulate hematopoietic stem/progenitor cells through the production of specific paracrine growth factors [21]–[24]. Kobayashi et al. demonstrated that the endothelial cells modulates reconstitution of hematopoietic stem/progenitor cells through the modulation of angiocrine factors with Akt-mTOR-activated endothelial cells supporting the self-renewal and expansion of hematopoietic stem/progenitor cells [24]. Recent studies also demonstrated endothelial cells regulate stem/progenitor cell niche in the central nervous system, and adipose tissues [25], [26]. Regarding the corneal epithelium, there is an indirect evidence of vascular niche that limbal niche cells have the angiogenesis potential and prevent corneal epithelial stem/progenitor cells differentiation [27].

In conclusion, we have shown that oral mucosal epithelial cell sheets can survive and contain putative epithelial stem/progenitor cells after transplantation. Because stem/progenitor cell maintenance in transplanted cell sheets is the key for a good prognosis, the results of our study are a first step toward understanding the behavior of these precursor cells in transplanted tissue.
